# Using stable-hydrogen isotopes to reveal immigration in an Arctic-breeding songbird population

**DOI:** 10.1186/s40462-016-0081-x

**Published:** 2016-06-15

**Authors:** Franz Bairlein, D. Ryan Norris, Christian C. Voigt, Erica H. Dunn, David J. T. Hussell

**Affiliations:** Institute of Avian Research “Vogelwarte Helgoland”, An der Vogelwarte 21, 26386 Wilhelmshaven, Germany; Department of Integrative Biology, University of Guelph, Guelph, N1G 2W1 ON Canada; Leibniz Institute for Zoo and Wildlife Research, Alfred-Kowalke-Str. 17, 10315 Berlin, Germany; Environment Climate Change Canada, National Wildlife Research Centre, Carleton University, 1125 Colonel By Drive, Ottawa, K1A 0H3 ON Canada; Ontario Ministry of Natural Resources, 2140 East Bank Drive, Peterborough, K9J 7BS ON Canada

**Keywords:** Immigration, Dispersal, Stable isotopes, Northern wheatear, *Oenanthe oenanthe*

## Abstract

**Background:**

Knowledge of immigration and emigration rates is crucial for understanding of population dynamics, yet little is known about these vital rates, especially for arctic songbirds. We estimated immigration in an Arctic population of northern wheatears on Baffin Island, Canada, by the use of stable hydrogen isotopes in tail feathers (δ^2^H_K_). We assumed that δ^2^H_K_ values of juvenile (hatch-year) feathers grown at the breeding grounds were representative of the local population, while those of breeding adults were indicative of where they grew their feathers during their post-breeding molt the previous year. The extent to which adult isotope values differ from those of juveniles provides an estimate of the minimum level of immigration into the breeding population.

**Results:**

Mean δ^2^H_K_ values did not differ in juvenile birds between years. Breeding adult birds did not differ significantly in mean δ^2^H_K_ values compared to juveniles but did differ in their respective standard deviations, reflecting a significantly wider range of isotopic signatures in adults than in juveniles. Thirty-eight percent of the δ^2^H_K_ values in adults were greater ± 2 SD of the mean δ^2^H_K_ values of juveniles, suggesting that at least 38 % of the breeding adults were of non-local origin, thus immigrants from elsewhere.

**Conclusions:**

Although the use of stable isotopes has limitations, the use of stable-hydrogen isotopic markers has the potential to contribute valuable information towards understanding immigration rates in bird populations. In our study, hydrogen isotope measurements of the feathers of northern wheatears indicated a high rate of immigration into the breeding population, which is consistent with low return rates of banded breeding adults as well as implying high emigration rates of local breeders.

**Electronic supplementary material:**

The online version of this article (doi:10.1186/s40462-016-0081-x) contains supplementary material, which is available to authorized users.

## Background

Dispersal is widely regarded as a key factor driving population dynamics and rate of gene flow [1–6]. Despite its importance, acquiring robust data on immigration and emigration rates has been extremely challenging. Mark-recapture approaches are often used for indirect estimation (e.g., [7–12]), but they require large sample sizes over multiple years and are labor intensive. Directly tracking movements using radio telemetry is also labor intensive and limited by the distribution of receivers across the landscape (e.g., [13]). Larger-scale marking schemes have recorded some long-distance dispersal events [3] but are still limited in their ability to record shorter distance movements, and detection rates suffer from many biases. Molecular markers have been effectively used to generate quantitative estimates of dispersal [14–16], although this approach relies on the presence of population-specific genetic signatures.

An alternative approach to estimating avian dispersal rates is to make use of stable isotopes [17–23]. Individuals incorporate a local isotopic signature through their diet [24, 25], and that signature is incorporated into growing feathers. Because feathers are metabolically inactive, they retain the isotopic signature from the location where they were grown [26]. Some stable isotopes, such as hydrogen [27], vary geographically and can, therefore, be used to estimate the location of feather growth, regardless of where or when the feather is sampled [19, 28–31]. Adult songbirds typically undergo post-breeding molt at or near their breeding sites, such that feathers sampled during the next breeding season carry the signature of the previous year’s breeding location. Feathers of juvenile birds have the local isotopic signature, so comparing juvenile and adult signatures can indicate amount of immigration.

We used this approach to estimate immigration rates of adult northern wheatears (*Oenanthe oenanthe*) in a breeding population located in Iqaluit, Nunavut, Canada. The northern wheatear is a small passerine of Old World origin that breeds in hot and cold deserts and open country throughout Europe and Asia and extends its breeding range eastward into western North America in Alaska and the Yukon, and westward from Europe into Iceland, Greenland and the eastern Canadian arctic [32]. All populations winter in sub-Saharan Africa, with western Arctic breeders migrating west to eastern Africa and the eastern Arctic population migrating across the Atlantic Ocean to winter in western Africa [33].

We assumed that the stable-hydrogen isotope values of juvenile feathers grown in July and August were representative of the local population, while those of breeding adults were indicative of where they grew their feathers during their post-breeding molt the previous year. The extent to which adult isotope values differ from those of juveniles provides an estimate of the minimum level of immigration into the breeding population and, by implication, the probable minimum level of emigration of our banded adults out of the population.

## Methods

### Study site, species and sampling

Our study area comprised about 20 km^2^ adjacent to the north shore of Frobisher Bay, Baffin Island, including the city of Iqaluit, Nunavut, Canada (63.7 N, 68.5 W) and extending about 3.5 km to the NW and 7 km to the SE from the city center. Northern wheatears breed throughout this coastal area, but are not found further inland.

In Iqaluit, northern wheatears arrive in early to mid-May and nest in natural rock crevices and under boulders, as well as in similar man-made sites such as rock walls. Most nest sites are inaccessible to humans. Six to ten eggs are laid starting as early as the last few days of May or as late as late June, but a few late or second clutches are started in the first half of July, some by adults that are already starting to molt [34]. The females incubate the eggs and both adults provision the nestlings. Fledging occurs mainly in July and the young are independent in August.

We searched the study area for breeding pairs, nests and fledged broods. Nests are difficult to find during the incubation stage, but relatively easy once the adults start to bring food to the nestlings. The number of person-days of searching effort varied from year-to-year, with greater effort in 2009–2011 when we equipped adults with light-sensitive archival data loggers and searched for their return [33]. During the nestling stage, adults were trapped at or near their nest sites with spring-loaded tent or bow nets baited with meal worms or with a “walk-in” trap placed in the entrance of the nest cavity. Each captured adult was marked with a unique combination of a standard numbered aluminum band and two or three color bands (Avinet Inc., Dryden, New York), which allowed us to recognize those individuals without recapturing them. All banded birds were aged and sexed by plumage where possible [32]. Young-of-the-year (HY for Hatch Year) could not be sexed, and adult females could not be split into age groups and, hence, were all classed as AHY (After Hatch Year). Males were classed as SY (Second Year, i.e. yearlings) or ASY (After Second Year), combined as AHY for certain analyses.

From 2009 to 2013, we sampled the 2nd outermost tail feather of breeding adult birds, and in 2010 and 2011 the same feather of recently fledged fully-grown birds (Nunavut Wildlife Research Permits WL 2009–034, 2010–028, 2011–032, 2012–035, 2013–042 and Canadian Wildlife Service Permits NUN-SCI-09-03, −10−02, −11−03, −12-05, −13−01). Feathers were kept in individual envelopes until analysis.

### Stable hydrogen isotope analyses

Stable hydrogen isotope (δD) values of feathers were analysed at the stable isotope laboratory of the Leibniz Institute for Zoo and Wildlife Research, Berlin, Germany. A small section of feather tissue was clipped from the tip of the feather (350 ± 7 μg) and loaded into silver capsules (IVA Analysetechnik e.K. Meerbusch, Germany). We filled 96 port microtiter trays with silver capsules loaded with feather samples and laboratory keratin standards with known δ^2^H values for non-exchangeable hydrogen. Trays were allowed to equilibrate with ambient air over more than 7 days. Afterwards, trays were placed in a drying oven over 1 day at 50 °C. Loaded capsules were then transferred to a zero blank autosampler (Costech Analytical Technologies Inc., Cernusco, Italy) above the elemental analyzer (HT elemental analyzer HEKAtech GmbH, Wegberg, Germany). For at least 1 h before combustion, samples were flushed in the autosampler with chemically pure helium (Linde, Leuna, Germany). We used a Delta V Advantage isotope ratio mass spectrometer (ThermoFischer Scientific, Bremen, Germany) that was connected via an interface (Finnigan Conflo III, ThermoFisher Scientific, Bremen, Germany) with the elemental analyzer. Reference H_2_ gases were calibrated against international standards (IAEA NBS 22 and IAEA-CH-7). We used the comparative equilibration method [35] to account for the amount of exchangeable hydrogen in feather keratin. We used three laboratory standards that covered the range of expected δD values in our samples. These standards were also used to determine the δD of non-exchangeable hydrogen [35, 36]. The stable hydrogen isotope ratios of the non-exchangeable hydrogen (mean ± s.d.) of the standards were: −133.6 ± 1.2 ‰, −109.1 ± 1.2 ‰ and −87.2 ± 1.0 ‰. In the sequential order of one autorun, keratin standards were placed at positions 1–6 (3 standards of 2 replicates) and at the 9th–11th position (3 standards). Hereafter, we refer to δ^2^H_K_ as the stable hydrogen isotope ratios of non-exchangeable hydrogen in feather keratin.

### Statistics

All statistics were performed using IBM SPSS Statistics 21. One-way ANOVA with post-hoc Tukey HSD-test was used to test differences between groups and Levene’s test to test for homoscedasticity. Prior to analysis, normality was tested using Kolmogorov-Smirnov-test statistics. None of the samples deviated significantly from normality. To estimate immigration rate we took the percentage of δ^2^H_K_ values in adults (AHY) that lie outside the ± 2 SD range of the distribution of δ^2^H_K_ values of the juveniles. The ± 2 SD range is equivalent to 95.4 % of the values around the mean [37].

## Results

### Banding and returns

Over the 8 years, 2007–2014, we determined whether or not the adults were already banded at almost 200 nest cavities, meaning a very high sampling effort. Of 204 banded adults ten returned in a later year (5 males and 5 females), representing 4.9 % of the banded adults from preceding years. Of 75 juveniles one returned (1.3 %), but none of 131 nestlings.

### Feather isotopes

Samples were collected from a total of 34 HY birds (2010: 18; 2011: 16) and 174 adults of both sexes (2009: 27; 2010: 46; 2011: 52; 2012: 20, 2013: 29). Of the 174 adults, 10 (2010: 2; 2011: 4; 2012: 3; 2013: 1) had δ^2^H_K_ values of higher than −100 ‰, which was very likely due to replacement of arbitrarily lost tail feathers at a far-distant non-breeding location [33]. Therefore, we excluded these values from any further analysis. For the remaining sample sizes see Additional file 1: Table S1.

**Fig. 1:**
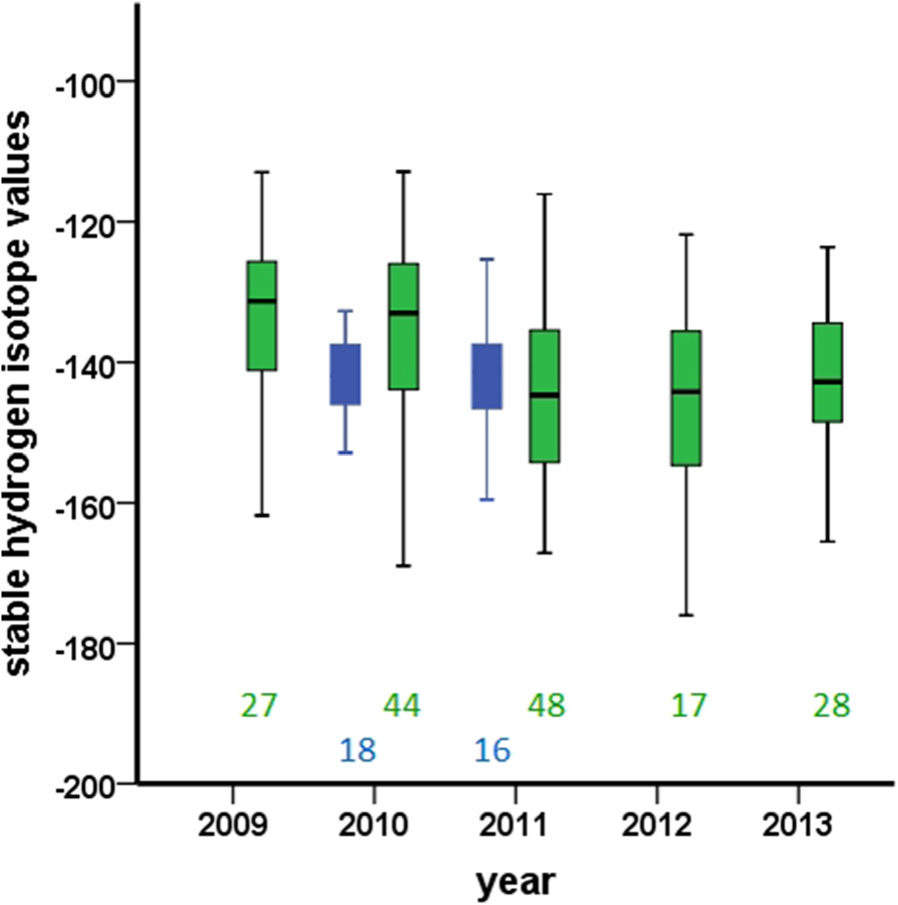
Annual stable-hydrogen isotope values of tail feathers (δ^2^H_K_) in Hatch Year (*HY, blue boxes*) and After Hatch Year (AHY, *green*; age/sex classes combined) of northern wheatears at Baffin Island, Canada. The boxplots show the median value (*horizontal band inside the box*), the first and third quartiles, and the whiskers give the minimum and maximum of all of the data excluding outliers. The figures at the x-axis show sample sizes. For statistics see text

There was no significant difference in mean δ^2^H_K_ between the 2 years in HY birds (Fig. 1; ANOVA, F_1,36_ = 0.138; *p* = 0.71). We assumed that these values were representative of δ^2^H_K_ signatures of feathers grown in our study area in Iqaluit and results for the 2 years were combined for further analysis.

Adult birds consisted of SY and ASY males and AHY females (for which age classes are indistinguishable in the field). For all adults combined, mean δ^2^H_K_ differed among years (ANOVA, F_4,159_ = 5.47; *p* < 0.001), with 2009 and 2010 showing significantly higher δ^2^H_K_ values than the other years (Fig. 1; post-hoc Tukey-HSD-test, *p* = 0.006 and 0.017, respectively). However, there were no significant differences in mean δ^2^H_K_ among adult age and sex classes (ANOVA, F_4,164_ = 0.337; *p* = 0.853), or in their respective standard deviations (Levene’s test; *p* = 0.936). We therefore combined all non-HY sexes into one group (AHY) for comparison with HYs.

**Fig. 2:**
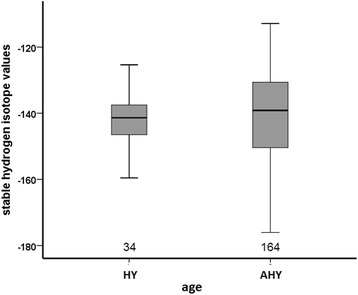
Boxplots of stable-hydrogen isotope values of tail feathers (δ^2^H_K_) of first-year birds (HY) and adult birds (AHY; all adult males and females combined). The figures at the x-axis give sample sizes

HY and AHY birds did not differ significantly in average δ^2^H_K_ values (ANOVA, F_1,198_ = 0.415; *p* = 0.520) but did differ in their respective standard deviations (Fig. 2; HY: −141.7 ± 6.7 ‰, *n* = 34; AHY: −140.2 ± 13.2 ‰, *n* = 164; Levene’s test: *p* < 0.001), reflecting a significantly wider range of δ^2^H_K_ in adults than in juveniles. In order to address whether the wider range in AHY birds is due to the larger sample size we randomly selected 100 times 34 cases from all the 164 AHY cases. Neither average mean nor average standard deviation of the repeats did differ from the entire data set (−140.4 ± 12.9 ‰ vs −140.2 ± 13.2 ‰). Therefore, we are confident having used the entire data set.

We had repeated measures from six adult birds that returned in the year after first capture. Stable-hydrogen isotope values in feathers averaged −140.5 ± 7.7 ‰ (SD) for returning birds, compared with −130.5 ± 7.6 ‰ in the first year of capture at Iqaluit. Importantly, the mean δ^2^H_K_ value of AHY birds at return was almost identical to the mean value for HY birds that grew their feathers at Iqaluit (−141.7 ± 6.7 ‰), which is consistent with the expectation that δ^2^H_K_ values of AHY and HY feathers are similar if grown at the same place. SD at first capture of these six birds was significantly lower than that for all AHY birds (7.7 *vs* 13.2 ‰; Levene’s test; *p* < 0.001) but similar to HY birds (Levene’s test; *p* > 0.05), revealing that returning breeders had a previous history of being more site faithful than is typical.

### Immigration rate

**Fig. 3:**
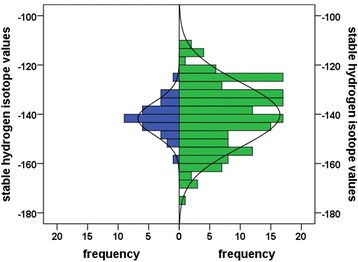
Frequency distribution of hydrogen isotope values (δ^2^H_K_) of tail feathers of HY (*blue*; *n* = 34) and AHY (*green*; *n* = 164) northern wheatears at Baffin Island, Canada. Curves: Fitted normal distribution

Thirty-eight percent of the δ^2^H values in adults were greater ± 2 SD of the mean δ^2^H values of juveniles (Fig. 3), suggesting that at least 38 % of the breeding adults were of non-local origin, thus immigrants from elsewhere. Having a closer look and comparing within a given year only (HY δ^2^H_K_ values from 2010 vs AHY δ^2^H_K_ from 2011 and HY δ^2^H_K_ values from 2011 vs AHY δ^2^H_K_ values from 2012), we found immigration rates of at least 40 and 28 %, respectively, suggesting that there was some annual variation in immigration rates as well.

## Discussion

Knowledge of immigration and emigration rates is crucial for full understanding of population dynamics, yet we know little about those rates in songbirds, especially arctic birds. In a German population of blackcaps *Sylvia atricapilla* in which all breeding adults and all fledged young were color-banded, 56 % of the breeding birds in the subsequent year were immigrants from other populations [7]. Other studies estimated immigration rates solely of yearling birds. In a Swedish population of collared flycatchers *Ficedula albicollis*, estimated immigration rates of yearling birds in unmanipulated control plots was ca 16 % [11]. In yellow-headed blackbirds *Xanthocephalus xanthocephalus*, Ward [12] recorded a proportion of yearling males of 14 % in Illinois and 24 % in South Dakota, respectively, and he cited several other North American studies of songbirds with annual percentages of yearling males ranging between 16 and 65 %.

For northern wheatears, our minimum estimate for all immigrants was at least 38 %, so at least 40 of the 104 adult males we captured were immigrants. Hatch-year birds made up 56.7 % of captured males, so 23 (about 22 %) of immigrant males were yearlings. These estimates fall within the range of values for temperate songbirds.

There is limited and often anecdotal data on breeding site fidelity of arctic songbirds. In a 6-year study of Lapland longspurs (*Calcarius lapponicus*) at Barrow, Alaska cumulative return of banded birds for all years yield 11.6 % for males and 23.5 %) for females [38]. At Sarcpa Lake, Nunavut, 47 of 86 (55 %) adult Lapland longspurs were detected as returnees in a subsequent year and 31 adults from an unknown number banded at McConnell River, Nunavut, returned the next year [39]. In a 3-year study of Eastern yellow wagtails (*Motacilla tschutschensis*) at Cape Romanzof, Yukon Delta National Wildlife Refuge, Alaska, at least 12 of 23 (52 %) banded males returned in the following year but none of 23 banded females returned, for an overall adult return rate of 26 % [40]. Fragmentary data on snow buntings (*Plectrophenax nivalis*) appear to indicate considerably lower breeding site fidelity: at Sarcpa Lake, Nunavut, 2 of 9 banded males and zero of 10 banded females returned the next year; on Devon Island, Nunavut, of 32 adults banded over a 3-year period, one male and two females returned the following year [41]; and at Iqaluit, Nunavut, only two (1 male and 1 female) of 22 adults fitted with geolocators in 2013 were found in 2014 (DJTH, pers. obs.). These figures suggest about 10 % return rate for adult snow buntings; still twice the rate of 5 % we found for northern wheatears by banding.

Although the use of stable isotopes provides a promising approach for estimating immigration and emigration in a population [17, 18, 20–23], there are several potential limitations to this approach. First, in most landscapes, stable isotopes do not provide the level of spatial accuracy to detect short distance (~ <100 km) movements, even when multiple isotopes are combined [23]. Thus, in our case, stable isotopes could underestimate the true number of immigrants. Second, our approach relies on population-specific values from known-origin tissues of a relatively large sample. Acquiring known-origin tissue can involve intensive fieldwork, as it typically requires locating and monitoring nests or catching juveniles soon after they leave the nest. A less intensive approach would be to characterize local δ^2^H distribution based on interpolated precipitation values [27] and then offset this distribution with a diet-tissue discrimination factor [29, 30]. We chose not to take this approach because we did not have an estimated diet-tissue discrimination factor for Arctic-breeding populations of this species or an ecologically similar species. However, obtaining such an estimate would, in theory, also allow us to assign the geographic origin of immigrants with a certain degree of confidence. Third, different diets of juveniles versus adult birds could have influence on the isotopic signatures of feathers. However, there is no evidence that adults and juvenile wheatears differ in their diets at the breeding grounds (e.g. [42–44]). Metabolism is certainly different in growing birds as compared to adults. But Storm-Suke et al. [45] provide experimental evidence in quail that metabolic rate does not influence diet-tissue discrimination in hydrogen isotope values. Moreover, the consistency in average similarity in the isotopic signatures between juvenile and adult wheatear tail feathers hints on no substantial effects of age.

Immigration and emigration are crucial factors driving demography and dynamics of bird populations but not much is known about their magnitude or their annual variation. The use of stable isotopes may help fill this gap. In our study, isotope measurements of the feathers of northern wheatears indicated a high rate of immigration into the breeding population, which is consistent with low return rates of banded breeding adults as well as implying high emigration rates of local breeders. If emigration of adults is high throughout their breeding lives, calculations of apparent survival may be inaccurate or impossible, and may not closely reflect true survival. However, in songbirds with high site-faithfulness once they become breeders [1], apparent survival of adult birds as revealed by return rates may not be far off of true survival.

## Additional file

Additional file 1: Table S1. Sample sizes. Abbreviations: HY = hatch-year, SY = second year, AHY = after hatch-year, ASY = after second year. (XLSX 11 kb)

## References

[1] Greenwood PJ, Harvey PH (1982). The natal and breeding dispersal of birds. Ann Rev Ecol Syst.

[2] Lebreton JD, Clobert J (1991). Bird population studies.

[3] Paradis E, Baillie SR, Sutherland WJ, Gregory RD (1998). Patterns of natal and breeding dispersal in birds. J Anim Ecol.

[4] Sutherland GD, Harestad AS, Price K, Lertzman KP. Scaling of natal dispersal distances in terrestrial birds and mammals. Conserv Ecol. 2000;4:16. [online] URL: http://www.consecol.org/vol4/iss1/art16.

[5] Matthysen E (2005). Density-dependent dispersal in birds and mammals. Ecography.

[6] Nevoux M, Arlt D, Nicoll M, Jones C, Norris K (2013). The short- and long-term fitness consequences of natal dispersal in a wild bird population. Ecol Lett.

[7] Bairlein F (1978). Über die Biologie einer südwestdeutschen Population der Mönchsgrasmücke (*Sylvia atricapilla*). J Ornithol.

[8] Marquiss M, Newton I (1982). A radio-tracking study of the ranging behaviour and dispersion of European Sparrowhawks *Accipiter nisus*. J Anim Ecol.

[9] Haas CA (1998). Effects of prior nesting success on site fidelity and breeding dispersal: An experimental approach. Auk.

[10] Newton I (2001). Causes and consequences of breeding dispersal in the Sparrowhawk *Accipiter nisus*. Ardea.

[11] Doligez B, Danchin E, Clobert J (2002). Public information and breeding habitat selection in a wild bird population. Science.

[12] Ward M (2005). The role of immigration in the decline of an isolated migratory bird population. Conserv Biol.

[13] Schaub M, Aebischer A, Gimenez O, Berger S, Arlettaz R (2010). Massive immigration balances high anthropogenic mortality in a stable eagle owl population: Lessons for conservation. Biol Conserv.

[14] Rannala B, Mountain JL (1997). Detecting immigration by using multilocus genotypes. Proc Natl Acad Sci U S A.

[15] Keller LF, Jeffery KJ, Arcese P, Beaumont MA, Hochachka WM, Smith JNM, Bruford MW (2001). Immigration and the ephemerality of a natural population bottleneck: evidence from molecular markers. Proc R Soc Lond B.

[16] Pruett CL, Arcese P, Chan YL, Wilson AG, Patten MA, Keller LF, Winker K (2008). The effects of contemporary processes in maintaining the genetic structure of western song sparrows (*Melospiza melodia*). Heredity.

[17] Graves GR, Romanek CS, Rodriguez NA (2002). Stable isotope signature of philopatry and dispersal in a migratory songbird. Proc Natl Acad Sci U S A.

[18] Hobson KA, Wassenaar LI, Bayne E (2004). Using isotopic variance to detect long-distance dispersal and philopatry in birds: an example with ovenbirds and American redstarts. Condor.

[19] Hobson KA (2005). Stable isotopes and the determination of avian migratory connectivity and seasonal interactions. Auk.

[20] Powell LA, Hobson KA (2006). Enriched feather hydrogen isotope values for Wood Thrushes sampled in Georgia, USA, during the breeding season: implications for quantifying dispersal. Can J Zool.

[21] Girvan MK, Jones J, Norris DR, Barg JJ, Kyser TK, Robertson RJ (2007). Long-distance dispersal patterns of male Cerulean Warblers (*Dendroica cerulean*) measured by stable-hydrogen isotopes. Avian Conserv Ecol.

[22] Van Wilgenburg SL, Hobson KA, Brewster KR, Welker JM (2012). Assessing dispersal in threatened migratory birds using stable hydrogen isotope (δD) analysis of feathers. Endanger Species Res.

[23] Haché S, Hobson KA, Bayne EM, Van Wilgenburg SL, Villard M-A (2014). Tracking natal dispersal in a coastal population of a migratory songbird using feather stable isotope (d2H, d34S) tracers. PLoS One.

[24] Cormie A, Scwarcz H, Gary J (1994). Relation between hydrogen isotopic ratios of bone collagen and rain. Geochim Cosmochim.

[25] Langin K, Reudink MW, Marra PP, Norris DR, Kyser TK, Ratcliffe LM (2007). Hydrogen isotopic composition of known-origin migratory birds: implications for geographic assignment. Oecologia.

[26] Hobson KA (1999). Tracing origins and migration of wildlife using stable isotopes: a review. Oecologia.

[27] Bowen GJ, Wassenaar LI, Hobson KA (2005). Global application of stable hydrogen and oxygen isotopes to wildlife forensics. Oecologia.

[28] Norris DR, Marra PP, Bowen GJ, Ratcliffe LM, Royle JA, Kyser TK (2006). Migratory connectivity of a widely distributed songbird, the American redstart (*Setophaga ruticilla*). Orn Monogr.

[29] Hobson KA, Wassenaar LI (2008). Tracking animal migration with stable isotopes.

[30] Hobson KA (2010). Isotopic ornithology: a perspective. J Ornithol.

[31] Hobson KA, Van Wilgenburg SL, Dunn EH, Hussell DJT, Taylor PD, Collister DM (2015). Predicting origins of passerines migrating through Canadian migration monitoring stations using stable-hydrogen isotope analyses of feathers: a new tool for bird conservation. Avian Conserv Ecol.

[32] Kren J, Zoerb AC. Northern Wheatear (*Oenanthe oenanthe*). In: A. Poole, F. Gill, editors. The Birds of North America, No. 316. The Academy of Natural Sciences, Philadelphia, PA, and The American Ornithologists’ Union, Washington, D.C.; 1997.

[33] Bairlein F, Norris DR, Nagel R, Bulte M, Voigt CC, Fox JW, Hussell DJT, Schmaljohann H (2012). Cross-hemisphere migration of a 25-gram songbird. Biol Lett.

[34] Hussell DJT, Bairlein F, Dunn EH (2014). Double-brooding by the Northern Wheatear on Baffin Island. Arctic.

[35] Wassenaar LI, Hobson KA (2003). Comparative equilibration and online technique for determination of nonexchangeable hydrogen of keratins for use in animal migration studies. Isot Environ Health Stud.

[36] Wassenaar LI, Hobson KA (2000). Stable-carbon and hydrogen isotope ratios reveal breeding origins of red-winged blackbirds. Ecol Appl.

[37] Sokal RR, Rohlf FJ (1995). Biometry.

[38] Custer TW, Pitelka FA (1977). Demographic features of a Lapland Longspur population near Barrow, Alaska. Auk.

[39] Hussell DJT, Montgomerie RD, Poole A, Gill F (2002). Lapland Longspur (*Calcarius lapponicus*). The Birds of North America, No. 656.

[40] Renner HM, McCaffery BJ (2008). Demography of Eastern Yellow Wagtails at Cape Romanzof, Alaska. Wilson J Ornithol.

[41] Lyon BE, Montgomerie RD. Snow Bunting and McKay’s Bunting (*Plectrophenax nivalis* and *Plectrophenax hyperboreus*). In: A Poole, F Gill, editors. The Birds of North America, No. 198–199. The Academy of Natural Sciences, Philadelphia, and The American Ornithologists’ Union, Washington, D.C.; 1995.

[42] Menzel H. Der Steinschmaetzer. Wittenberg: Ziemsen; 1964.

[43] Conder P. The Wheatear. London: Christopher Helm; 1989.

[44] van Oosten HH, van den Burg AB, Versluijs R, Siepel H (2014). Habitat selection of brood-rearing Northern Wheatears *Oenanthe oenanthe* and their invertebrate prey. Ardea.

[45] Storm-Suke A, Norris DR, Wassenaar LI, Chin E, Nol E (2012). Hydrogen Isotopes in Proteinaceous Tissue: Experimental Results Using Japanese Quail. Physiol Biochem Zool.

